# Supplementary material for: The Influence of Parathyroidectomy on Osteoporotic Fractures in Kidney Transplant Recipients: Results from a Retrospective Single-Center Trial
Source: J Clin Med. 2022 Jan 27;11(3):654. doi: 10.3390/jcm11030654 (PMC8836679; doi:10.3390/jcm11030654)
Supplement: Supplementary file 1 [file jcm-11-00654-s001.zip › jcm-1559807-supplementary.pdf]

Supplements:

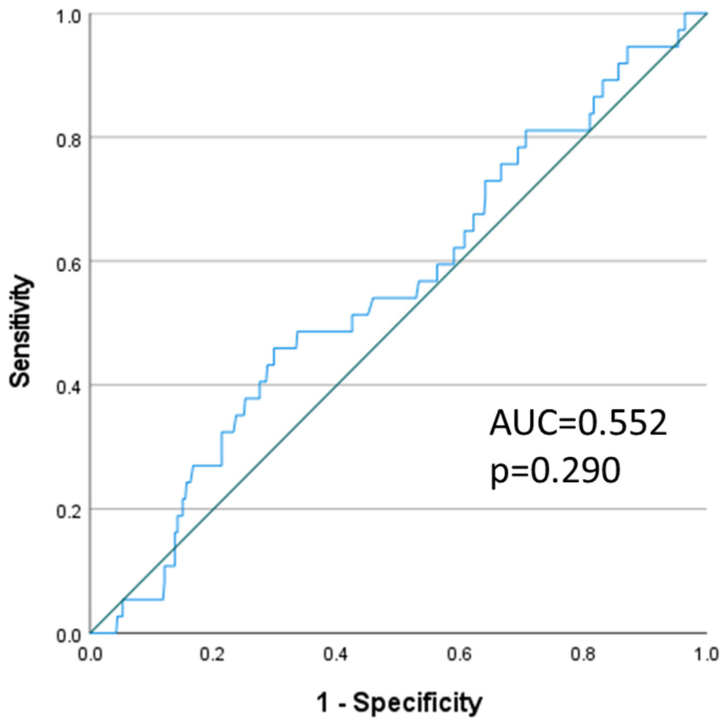

Figure S1. ROC-analysis for PTH levels three months after KTx and fracture occurrence. Based on this, we could not support a cut-off for PTH ≥ 130 pg/mL being associated with an increased fracture risk.
